# Microbial Colonization and Antibiotic Resistance Profiles in Chronic Wounds: A Comparative Study of Hidradenitis Suppurativa and Venous Ulcers

**DOI:** 10.3390/antibiotics14010053

**Published:** 2025-01-09

**Authors:** Florica Sandru, Elena Poenaru, Smaranda Stoleru, Andreea-Maria Radu, Alexandra-Maria Roman, Corina Ionescu, Aurelian Zugravu, Jafal Mugurel Nader, Livia-Cristiana Băicoianu-Nițescu

**Affiliations:** 1 Department of Dermatovenerology, “Carol Davila” University of Medicine and Pharmacy, 020021 Bucharest, Romania; 2 Dermatology Department, “Elias” University Emergency Hospital, 011461 Bucharest, Romania; 3 Medical Informatics and Biostatistics Discipline, “Carol Davila” University of Medicine and Pharmacy, 020021 Bucharest, Romania; 4 Pharmacology and Pharmacotherapy Discipline, “Carol Davila” University of Medicine and Pharmacy, 020021 Bucharest, Romania; 5 Anesthesia and Intensive Therapy Discipline, “Carol Davila” University of Medicine and Pharmacy, 020021 Bucharest, Romania

**Keywords:** hidradenitis suppurativa, venous ulcer, chronic wound, wound colonization, antibiotic resistance, *Staphylococcus aureus*, MRSA, *Pseudomonas aeruginosa*

## Abstract

**Background/Objectives**: Chronic wounds, including hidradenitis suppurativa (HS) and venous ulcers (VU), are commonly associated with complex microbial communities that may influence wound healing and treatment outcomes. Understanding microbial diversity and antibiotic resistance patterns is essential in order to optimize therapeutic strategies. This study aimed to investigate the microbial populations and antibiotic resistance profiles in HS and VU patients, comparing the prevalence of common pathogens and their antimicrobial resistance profiles. **Methods**: We conducted a cross-sectional analysis that included a total of 112 individuals (24 with the diagnosis of hidradenitis suppurativa and 88 diagnosed with venous ulcer). Wound swabs were cultured to identify bacterial species, and antibiotic resistance was assessed using a standard panel of antibiotics. Prevalence rates of key pathogens, such as *Staphylococcus aureus*, *Pseudomonas aeruginosa*, and *Enterococcus faecalis*, were compared between the two groups. Resistance patterns were analyzed using statistical methods to identify significant differences. **Results**: *Staphylococcus aureus* was the most common pathogen in both groups (45.8% in HS; 38.6% in VU), with a notable prevalence of methicillin-resistant *S. aureus* (MRSA). *Pseudomonas aeruginosa* was exclusively identified in VU patients (27.3%), while beta-hemolytic *Streptococcus* and *Corynebacterium amycolatum* were identified in HS cases only. Antibiotic resistance was moderate, notably in *S. aureus* and *Proteus mirabilis*, while one case of multidrug-resistant *Pseudomonas aeruginosa* was identified. **Conclusions**: This study highlights the distinctive microbial profiles and antibiotic resistance patterns in HS and VU chronic wounds. The predominance of *S. aureus* in both groups underscores the need for targeted therapies, while the absence of *P. aeruginosa* in HS wounds and the higher prevalence of other species emphasizes wound-specific microbial variations. These findings underscore the importance of personalized treatment strategies and continuous surveillance of antimicrobial resistance.

## 1. Introduction

A wound is defined as a disruption in the continuity of cellular, anatomical, and functional structures of living tissue, caused by various factors, such as physical, chemical, thermal, microbial, or immunological injury. This disruption typically begins with a breach in epithelial cohesion, which may extend to compromise the structural and functional stability of the underlying tissue. Chronic wounds are characterized by a prolonged healing process, often due to impaired angiogenesis, innervation, or cellular migration. These wounds, particularly those associated with cutaneous ulceration and systemic conditions such as autoimmune or inflammatory disorders, pose significant treatment challenges. Additionally, they are often colonized by antibiotic-resistant bacteria, requiring effective management of the underlying condition before considering definitive surgical intervention [1,2,3]. Skin and soft tissue infections frequently involve cellulitis, which is characterized by localized erythema, edema, and increased local temperature. This condition occurs due to bacterial entry through disruptions in the skin barrier, resulting in an inflammatory response [4,5,6]. Cellulitis predominantly affects middle-aged and elderly individuals, with various factors heightening the risk. Trauma-related disruption of the skin barrier, inflammatory skin conditions like eczema and psoriasis, edema from impaired lymphatic drainage or venous insufficiency, and obesity all contribute to susceptibility [7,8,9,10,11]. Studies showed that 92% of lymphedema-related hospital admissions are associated with cellulitis [12]. Immunosuppression, unnoticed skin breaks, pre-existing infections, and close contact with individuals carrying methicillin-resistant *Staphylococcus aureus* (MRSA) represent additional risks for purulent infections [13]. Complications arising from cellulitis and abscesses can lead to serious systemic conditions, such as bacteremia, endocarditis, septic arthritis, osteomyelitis, metastatic infections, sepsis, and toxic shock syndrome. These severe outcomes underscore the importance of recognizing and addressing predisposing factors, as outlined, to prevent the escalation of localized infections into more life-threatening conditions [4]. The primary cause of cellulitis is beta-hemolytic streptococci, particularly group A *Streptococcus*, with *Staphylococcus aureus*, including methicillin-resistant strains, being a less common but notable pathogen. In some cases, Gram-negative bacilli may also be implicated [14,15,16,17].

Chronic venous insufficiency, which leads to recurrent edema, is a key factor predisposing individuals to recurrent cellulitis, especially when accompanied by venous leg ulcers (VLUs). VLUs, the most common chronic wound, affecting approximately 4% of the population, typically occur in the lower extremities and are associated with high recurrence rates, prolonged disability, and psychosocial burden [18]. These ulcers, which vary in size and severity, can produce excessive exudate, leading to skin irritation and further complications like cellulitis [19,20,21,22,23,24]. Venous stasis ulcers constitute over half of all chronic lower-limb wounds. These wounds are often colonized by pathogens such as *Staphylococcus aureus*, *Pseudomonas aeruginosa*, and β-hemolytic streptococci, which hinder healing by inducing inflammation. Bacterial presence attracts leukocytes, leading to an increase in inflammatory cytokines, proteases, and reactive oxygen species (ROS), thus perpetuating inflammatory cycles that impede wound repair [25]. Signs of infection, such as localized heat, erythema, or fever, require prompt diagnosis and treatment in order to prevent cellulitis from progressing to more severe complications, such as bacteremia or sepsis [26].

Hidradenitis suppurativa (HS) is a chronic inflammatory skin condition that primarily affects regions characterized by a high density of hair follicles, with prevailing hypotheses suggesting that it arises from follicular occlusion [27]. European studies, which include undiagnosed cases, typically estimate a prevalence of 1% or higher, indicating that the condition is relatively common [28]. The disease pathogenesis is likely multifactorial, with genetic predisposition, cellular and immune factors, nutrition, obesity, and smoking—as a risk factor for many chronic conditions, including psoriasis—all playing potential roles [29,30]. The clinical manifestations of HS vary widely, ranging from inflamed nodules and abscesses to draining sinus tracts, often resulting in significant pain, malodor, and scarring, which adversely affect the quality of life [31,32]. The involvement of bacteria in HS remains a subject of contention; early lesions are generally sterile, whereas older or ruptured lesions may be colonized by various microorganisms, including staphylococci, streptococci, and other facultative anaerobes. Although these bacteria may signify secondary infections or mere contaminants, some theories postulate that bacterial biofilms may exacerbate the chronic inflammatory response associated with HS [33]. In cases that are severe or inadequately managed, bacterial infections, including cellulitis, represent notable complications [34,35,36,37,38,39].

Bacterial resistance to antimicrobials can be either intrinsic or acquired. Intrinsic resistance is a natural trait exhibited by nearly all members of a species, as seen in *Klebsiella pneumoniae’s* resistance to ampicillin, eliminating the need for susceptibility testing [40]. Acquired resistance, however, develops in response to antimicrobial exposure in previously susceptible bacteria, often through chromosomal mutations or horizontal gene transfer via plasmids, integrons, transposons, or transformation. Unlike intrinsic resistance, acquired resistance varies among isolates, making susceptibility testing essential for therapeutic decisions [41]. Resistance expression may be constitutive, occurring continuously, or inducible, triggered by specific agents; for example, third-generation cephalosporins can induce AmpC beta-lactamase production in some *Enterobacterales* [42]. Additionally, heteroresistance, a variable expression of resistance within bacterial subpopulations, poses challenges for detection, sometimes requiring susceptibility tests with higher inocula to identify resistant subpopulations accurately, as seen in vancomycin-intermediate *Staphylococcus aureus* [43]. The terms “susceptible”, “intermediate”, and “resistant” categorize microorganisms based on their responses to antimicrobial agents. Susceptible organisms are effectively inhibited or killed by antibiotics at standard dosing levels, while resistant organisms fail to respond even at concentrations exceeding safe or achievable levels in the body. The intermediate category reflects an uncertain response, where higher doses or optimal conditions might render the antibiotic effective [44]. These classifications are crucial for optimizing treatment and understanding overall resistance patterns within populations. Moreover, standardizing antimicrobial susceptibility testing methods and adopting harmonized evaluation criteria are essential for accurate classification. Such standardization enhances clinical decision making, supports resistance monitoring, and ensures consistency in reporting across laboratories and regions [44,45].

## 2. Results

### 2.1. Bacterial Profiles in Hidradenitis Suppurativa vs. Venous Ulcers

**Figure 1:**
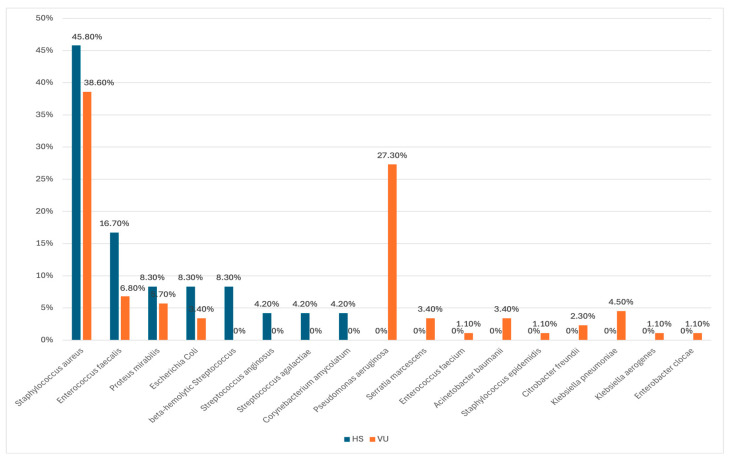
Percentual Frequency of Bacterial Species in Patients with Hidradenitis Suppurativa and Venous Ulcers. HS = Hidradenitis Suppurativa; VU = Venous Ulcer.

In our study, we identified a total of 112 patients, comprising 24 with hidradenitis suppurativa (HS) and 88 with venous ulcers (VU). The cohort included 66 male and 46 female patients, with ages ranging from 18 to 93 years and a mean age of 60.09 ± 18.49 years. In the HS subgroup, wound swab cultures revealed *Staphylococcus aureus* in 45.8% of cases, *Enterococcus faecalis* in 16.7%, and *Proteus mirabilis*, beta-hemolytic *Streptococcus* and *Escherichia coli* each in 8.3% of cases. Additionally, *Streptococcus anginosus*, *Streptococcus agalactiae*, and *Corynebacterium amycolatum* were each identified in 4.2% of cases. In the VU subgroup, the most frequently identified bacterial species were *Staphylococcus aureus* (38.6%), *Pseudomonas aeruginosa* (27.3%), *Enterococcus faecalis* (6.8%), and *Proteus mirabilis* (5.7%). Additional species and corresponding percentages are detailed in Figure 1. The comparison between the two subgroups revealed two statistically significant differences with a highly suggestive *p*-value of 0.001. *Pseudomonas aeruginosa* was notably more prevalent in the VU subgroup than in the HS subgroup (27.3% vs. 0%), while beta-hemolytic *Streptococcus* appeared more frequently in the HS subgroup compared to the VU subgroup (8.3% vs. 0%).

In regard to the sexes, in the (HS) subgroup, *Staphylococcus aureus* was the most prevalent pathogen in both females (60%) and males (42.1%), while *Proteus mirabilis* and *E. coli* were each isolated in 10.5% of males but completely absent in the female population. *Enterococcus faecalis* was identified in 20% of females and 15.8% of males, while *Streptococcus anginosus* and *Streptococcus agalactiae* were only found in males (5.3% each). Beta-hemolytic *Streptococcus* appeared in 20% of females and 5.3% of males, and lastly, *Corynebacterium amycolatum* was found only in males (5.3%). In the HS subgroup, there was no statistically significant difference in bacterial species distribution between male and female patients (*p* = 0.7325).

**Figure 2:**
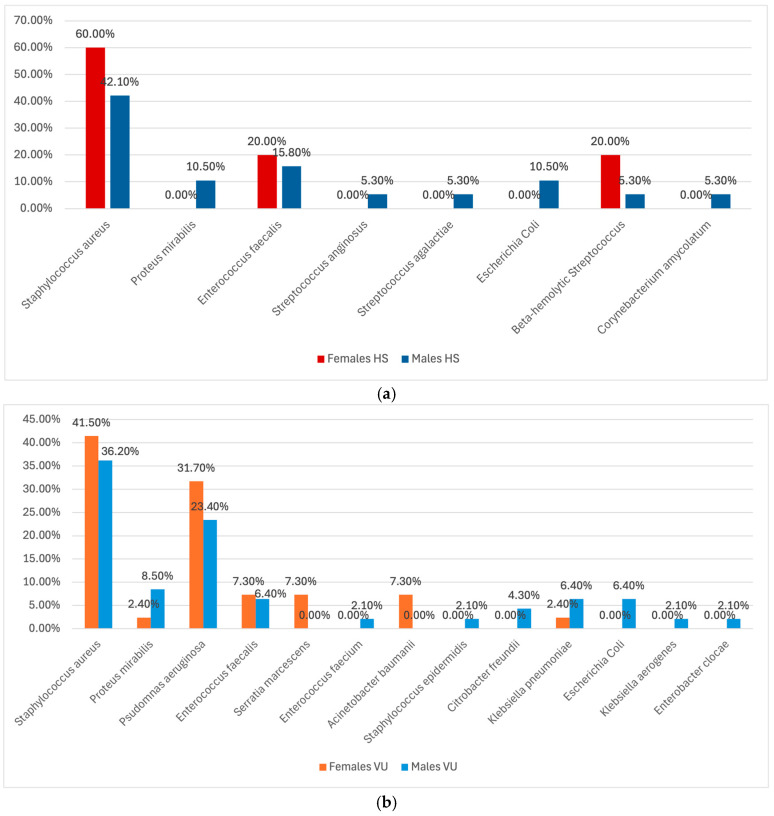
(**a**). Comparison of isolated bacterial strains between females and males in the HS subgroup. (**b**). Comparison of isolated bacterial strains between females and males in the VU subgroup. HS = Hidradenitis Suppurativa; VU = Venous Ulcer.

However, in the VU subgroup, a greater variety of bacterial species was identified among male patients (*p* = 0.023), with *Pseudomonas aeruginosa* being significantly more prevalent in females (31.7%) than males (23.4%). Moreover, in the VU subgroup, *Staphylococcus aureus* was the most commonly identified pathogen, affecting 41.5% of females and 36.2% of males. *Proteus mirabilis* was found in 2.4% of females and 8.5% of males, while *Serratia marcescens* appeared only in females (7.3%). *Acinetobacter baumannii* and *Staphylococcus epidermidis* were both identified only in males (0% in females), while other pathogens like *E. coli*, *Klebsiella pneumoniae*, and *Citrobacter freundii* were more common in males. These results are shown in Figure 2a,b.

### 2.2. Antibiotic Resistance in Key Isolates

In our study, we assessed the mean number of antibiotics to which the most frequently identified bacterial species were resistant. *Staphylococcus aureus* exhibited resistance to an average of 1.64 ± 1.708 antibiotics (median: 2.00), *Proteus mirabilis* to an average of 2.29 ± 1.380 (median: 2.00), *Pseudomonas aeruginosa* to an average of 0.88 ± 2.232 (median: 0.00), *Enterococcus faecalis* to an average of 0.10 ± 0.316 (median: 0.00), beta-hemolytic *Streptococcus* to an average of 1.00 ± 1.414 (median: 1.00), and *Escherichia coli* to an average of 0.80 ± 0.37 (median: 1.00) antibiotics. Results are listed in Table 1.

#### 2.2.1. *Staphylococcus aureus*

In this cohort, 19 cases of *Staphylococcus aureus* demonstrated no antibiotic resistance, including 4 cases in the hidradenitis suppurativa (HS) subgroup and 15 in the venous ulcer (VU) subgroup. Additionally, four cases—two from the HS subgroup and two from the VU subgroup—exhibited resistance to five or more antibiotics. All *Staphylococcus aureus* isolates showed full sensitivity (100%) to tigecycline, teicoplanin, and vancomycin. The antibiotic resistance profile further revealed that 32.6% of tested isolates were resistant to oxacillin, indicating the presence of MRSA strains, while the remaining 67.4% were oxacillin-sensitive. Moderate resistance rates were observed for clindamycin and erythromycin, with 53.5% and 55% resistance, respectively. Furthermore, in this study, the relationship between primary diagnosis (HS and VU, respectively) and MRSA isolates was analyzed to determine whether the primary diagnosis impacted resistance patterns. Among the 43 cases, 45.5% of isolates from the HS group were resistant to oxacillin compared to 28.1% of isolates from the VU group. Overall, 32.6% of all isolates were resistant. Statistical analysis revealed no significant association between primary diagnosis and oxacillin resistance (*p* = 0.290). These results indicate that the primary diagnosis (whether HS or VU) does not significantly influence the likelihood of oxacillin resistance in *S. aureus* isolates and thus the prevalence of MRSA, within this population. Results are shown in Table 2.

#### 2.2.2. *Proteus mirabilis*

In the analyzed cohort, all *Proteus mirabilis* isolates exhibited resistance to at least one of the following antibiotics: colistin, tigecycline, or tetracycline. Additionally, 57.1% of the isolates demonstrated resistance to multiple of the tested antibiotics, with ampicillin and gentamicin showing particularly high resistance rates—57.1% and 71.4%, respectively. The isolates demonstrated full susceptibility to amikacin (100%), ceftazidime (100%), ceftriaxone (100%), meropenem (100%), and ertapenem (100%). Mixed results were observed with co-trimoxazole, where 57.1% of isolates were susceptible, and 42.9% were resistant. Results can be observed in Table 3.

#### 2.2.3. *Pseudomonas aeruginosa*

In the studied population, *Pseudomonas aeruginosa* was identified in 24 out of 88 individuals with venous ulcers (27%) and in none of the 24 individuals with hidradenitis suppurativa. Among these cases, one isolate of *Pseudomonas aeruginosa* exhibited resistance to all tested antibiotics, including combination therapies such as piperacillin-tazobactam, ceftazidime-avibactam, ceftolozane-tazobactam, imipenem-relebactam, and meropenem-vaborbactam, with the exception of colistin and cefiderocol. In contrast, 14 isolates showed complete susceptibility. Resistance among *Pseudomonas aeruginosa* isolates was highest for oxacillin and ceftolozane-tazobactam, both at 100.0%, while susceptibility to gentamicin was at 100.0%. Susceptibility to amikacin and tobramycin was also high, at 95.2% and 94.4%, respectively. Mixed responses were noted with ceftazidime, where 85.7% of isolates were either intermediate or sensitive. Levofloxacin was not a preferable option for treating *P. aeruginosa* infections, as 47.4% of cases showed intermediate susceptibility, and 42.1% demonstrated complete resistance. Similarly, imipenem was also unsuitable, with 95.0% of cases exhibiting only intermediate susceptibility. Piperacillin-tazobactam demonstrated 81.0% intermediate effectiveness, and colistin had a full susceptibility rate of 100.0%. Results can be observed in Table 4.

#### 2.2.4. *Enterococcus faecalis*

In this population, we identified a total of 10 cases of *Enterococcus faecalis*, with 4 cases in the HS subgroup and 6 in the VU subgroup. Of these, 9 out of 10 cases were sensitive to all tested antibiotics, while only 1 out of the 6 cases in the VU subgroup exhibited resistance to a single antibiotic, specifically gentamicin. For *Enterococcus faecalis* isolates, the valid susceptibility was highest with ampicillin (100.0%), gentamicin (90.0%), and vancomycin (100.0%). In the tested isolates, 20.0% were fully susceptible to linezolid and ertapenem, while 30.0% were fully susceptible to teicoplanin and tigecycline. Additionally, all tested isolates demonstrated 100.0% susceptibility to amikacin, tobramycin, ceftazidime, ceftriaxone, levofloxacin, co-trimoxazole, meropenem, colistin, and ceftazidime-avibactam. Results are noted in Table 5.

#### 2.2.5. *Escherichia coli*

In the analyzed group, a total of five cases of *Escherichia coli* were identified, consisting of two cases in the HS subgroup and three in the VU subgroup. Regarding resistance profiles, 100.0% susceptibility rates were observed in gentamicin, co-trimoxazole, and piperacillin-tazobactam. *E. coli* isolates showed an 80.0% susceptibility to amoxicillin-clavulanate, while ampicillin exhibited a 40.0% susceptibility rate. Additionally, *E. coli* was fully susceptible (100.0%) to amikacin, tobramycin, ceftazidime, ceftriaxone, levofloxacin, meropenem, and ertapenem. Results are given in Table 6.

#### 2.2.6. Beta-Hemolytic *Streptococcus*

Two cases of beta-hemolytic *Streptococcus* were identified in the HS subgroup, while none were found in the VU subgroup. Of the two cases, one exhibited no antimicrobial resistance. The beta-hemolytic *Streptococcus* isolates showed 100.0% susceptibility to penicillin, erythromycin, co-trimoxazole, and rifampicin. However, tetracycline and clindamycin demonstrated mixed susceptibility, with 50.0% of *Streptococcus* isolates proving susceptible and 50.0% resistant. Results can be observed in Table 7.

## 3. Discussion

Our study provides valuable insights into the microbial populations associated with chronic wounds, particularly in patients with hidradenitis suppurativa (HS) and venous ulcers (VU). We observed patterns that align with findings from the literature but also revealed unique differences in microbial diversity and antibiotic resistance profiles.

Our results show that *Staphylococcus aureus* was the most frequently identified pathogen in both groups (45.8% in HS; 38.6% in VU). This aligns with Wysocki et al., who identified *S. aureus* as a dominant pathogen in venous ulcers, and Wolcott et al., who similarly found *S. aureus* to be a prominent chronic wound pathogen [46,47]. Furthermore, Wong et al. reported comparable prevalence rates for *S. aureus* (23.3%) in chronic wounds, further supporting our findings [48]. In line with our study, Katoulis et al. also found a high prevalence of *S. aureus* in HS wounds [49]. However, our observed prevalence of *S. aureus* (45.8%) in HS patients contrasts with the very high rates reported by Miller et al. for venous leg ulcers (up to 90%), suggesting that the prevalence of this pathogen might vary significantly depending on wound type and demographic factors [50]. Moreover, *Staphylococcus aureus*, particularly methicillin-resistant *S. aureus* (MRSA), is a major pathogen responsible for a wide range of infections, from mild skin conditions to severe diseases like pneumonia and endocarditis. The bacterium has developed resistance through various mechanisms, including β-lactamase production, efflux systems, and biofilm formation, which complicate treatment. Resistance to penicillin exceeds 90%, and the emergence of vancomycin-resistant *S. aureus* (VRSA) further limits therapeutic options. The global prevalence of MRSA is rising, particularly in developing countries where poor hygiene and overcrowded conditions contribute to the spread of community-acquired MRSA (CA-MRSA). In contrast, hospital-acquired MRSA (HA-MRSA) infections are declining in developed regions due to improved healthcare practices. However, the increasing prevalence of multidrug-resistant strains and the emergence of severe infections underscore the urgent need for continued surveillance and enhanced antimicrobial stewardship worldwide [51,52,53]. Similar to our results, MRSA, as reported in China, showed higher resistance to various antibiotics, including macrolides, quinolones, and aminoglycosides, while remaining highly sensitive to vancomycin and teicoplanin. Our study also found significant resistance to clindamycin and erythromycin, with resistance rates of 53.5% and 55%, respectively. These findings align with global trends, where MRSA strains generally exhibit moderate resistance to several antibiotics but remain responsive to vancomycin and teicoplanin [52].

In addition to their antimicrobial effects, antibiotics have significant anti-inflammatory properties, which are particularly relevant in managing hidradenitis suppurativa (HS). These properties extend beyond pathogen elimination, targeting the inflammatory pathways that exacerbate HS. Antibiotics are considered first-line treatment due to their antimicrobial, anti-inflammatory, and immunomodulatory effects, underscoring the importance of careful selection to balance efficacy with their role in modulating inflammation. However, the growing challenge of antimicrobial resistance, particularly related to biofilm production, highlights the need to refine treatment approaches and reassess the clinical value of swab-guided antibiotic therapy, warranting further evaluation in randomized clinical trials [54].

*Pseudomonas aeruginosa* is a major cause of severe hospital-acquired infections and poses a significant global health threat due to its high levels of antimicrobial resistance (AMR). Resistance mechanisms, including efflux pump overexpression, porin mutations, and β-lactamase production, hinder the effectiveness of multiple antibiotics, complicating treatment, especially in immunocompromised patients. The pathogen’s ability to acquire resistance genes through horizontal gene transfer further exacerbates the challenge. AMR in *P. aeruginosa* is a critical concern worldwide. Addressing this growing issue requires enhanced surveillance, improved healthcare infrastructure, and targeted treatment strategies to combat the spread of multidrug-resistant strains and protect public health [51,53,55,56].

A striking difference in our study was the absence of *Pseudomonas aeruginosa* in HS patients, while it was identified in 27.3% of VU patients. This aligns with findings from Bialasik, who also reported a higher prevalence of *P. aeruginosa* in venous leg ulcers [57]. Moreover, our study also aligns with findings by Rahim and Dowd et al., where *P. aeruginosa* was less prevalent in HS than in other chronic wound types, further supporting the hypothesis that microbial populations in HS may differ significantly from those in other wound types [58,59]. Notably, *Pseudomonas aeruginosa* was present in 31.7% of cases analyzed by Benzecry et al., suggesting that local factors might influence microbial populations in HS wounds [60]. Furthermore, our results resonate with those of Nikolakis et al., who found a greater prevalence of *P. aeruginosa* in venous ulcers than in HS wounds, potentially influenced by differences in wound environments and host factors [29]. In contrast, studies by Brook et al. and Jockenhöfer et al. identified *P. aeruginosa* across both VU and HS cases [61,62]. Our study highlights the need to consider such variations when diagnosing and treating chronic wounds.

The antimicrobial resistance (AMR) profiles of *Pseudomonas aeruginosa* in this study align with global concerns about the increasing resistance of this pathogen to multiple antibiotics. Resistance in our cohort was highest to oxacillin and ceftolozane-tazobactam, which is consistent with findings from studies in other regions, such as those in China, where resistance to carbapenems like imipenem and meropenem has fluctuated over the years. In China, resistance to imipenem dropped from 30.8% in 2010 to 22.1% in 2022, while meropenem resistance decreased from 25.8% to 17.6% in the same period, although carbapenem-resistant *P. aeruginosa* (CRPA) remained a concern, particularly in Zhejiang Province, where resistance reached 38.67% by 2017 [52]. Similarly, in the Arabian Gulf countries, *P. aeruginosa* demonstrated significant resistance to meropenem (10.3–45.7%) but remained largely susceptible to colistin [63]. Our study also observed high susceptibility to gentamicin, amikacin, and tobramycin, which contrasts with the findings of Telling et al., where resistance to gentamicin was present in 19.6% of isolates [64]. Additionally, the prevalence of cefiderocol non-susceptibility (CFDC-NS) in *P. aeruginosa* has been generally low in global studies but notably higher in carbapenem-resistant strains, highlighting the challenge of treating resistant infections with newer agents [65]. Collectively, these studies underscore the global variability in resistance patterns, emphasizing the urgent need for continuous regional surveillance and standardized testing to guide treatment strategies and combat the growing threat of multidrug-resistant *P. aeruginosa.*

Interestingly, our study identified beta-hemolytic *Streptococcus* and *Corynebacterium amycolatum* in the HS subpopulation only. This finding is consistent with Benzecry et al., who identified similar bacterial species in HS patients, though with differences in prevalence rates. Specifically, while we found beta-hemolytic *Streptococcus* in 8.3% of HS cases, Benzecry et al. reported *Streptococcus* spp. in approximately 5.3% of their cohort [60]. In contrast, Eriksson et al., found that *Streptococcus* species were more likely to colonize venous ulcers due to the unique inflammatory environment in these lesions [66].

In terms of antibiotic resistance, our findings show moderate resistance to multiple antibiotics among the most frequently identified pathogens. *S. aureus* exhibited resistance to an average of 1.64 ± 1.71 antibiotics, and *Proteus mirabilis* demonstrated resistance to 2.29 ± 1.38 antibiotics. This is in line with studies by Ayobami et al. and Bettoli et al., who noted higher resistance rates in MRSA and *P. aeruginosa* isolates in North America and Asia, respectively [67,68]. However, the resistance patterns in our study did not reach statistical significance, potentially due to the relatively small sample size or the inclusion of both sensitive and resistant strains in the analysis.

Notably, we found that 100% of the isolates tested were sensitive to tigecycline, teicoplanin, and vancomycin. These findings are consistent with reports from Wysocki et al., who also noted high sensitivity of chronic wound pathogens to these antibiotics, particularly in cases involving MRSA and multidrug-resistant strains [69]. Our study also observed moderate resistance to common antibiotics like erythromycin and clindamycin, which is consistent with findings by Hessam et al. regarding *S. aureus* resistance in HS infections [70].

In the HS group, we also observed a significant prevalence of *Enterococcus faecalis*, with 9 out of 10 cases showing sensitivity to all tested antibiotics. This shows that while *Enterococcus* species are commonly found in chronic wounds, they did not exhibit high resistance in the cohort we studied. This finding contrasts with studies in other regions, where *Enterococcus* species showed higher resistance profiles, especially in the context of polymicrobial infections [68,70].

Furthermore, our findings mirror the microbial diversity reported by Dowd et al., who highlighted *Staphylococcus aureus* and *Pseudomonas aeruginosa* as dominant pathogens in chronic wounds but also showed that Enterobacteriaceae, including *E. coli* and *Proteus mirabilis*, are common in venous ulcers [59]. The research conducted by Thomas et al. on wound microbiomes in oxygen-deficient environments further supports our observation of anaerobic conditions being more pronounced in HS lesions, which may contribute to the unique microbial profiles observed in these wounds [71].

The antimicrobial resistance profiles observed in our study align with global trends, particularly regarding *Escherichia coli*, *Enterococcus faecalis*, *Proteus mirabilis*, and *Streptococcus species*. For *E. coli*, our study found high susceptibility to gentamicin and co-trimoxazole, with moderate resistance to ampicillin, similar to the resistance patterns reported by Abayneh et al., who found high resistance rates to ampicillin (89%) and co-trimoxazole (83%) [72]. In a study conducted in China, *E. coli* also showed moderate resistance to ciprofloxacin (61.4%) and cefepime (25.1%) but remained highly susceptible to carbapenems, a trend reflected in our study where *E. coli* isolates were fully susceptible to meropenem and ertapenem, aligning with findings published by Luo et al. [52]. *Enterococcus faecalis* in our cohort exhibited a similar trend, with high susceptibility to ampicillin and vancomycin, contrasting with the growing global concerns of vancomycin-resistant enterococci (VRE), highlighted by Zhu et al. [73]. Our findings for *Proteus mirabilis* were consistent with the study conducted by Torrens et al., which showed complete susceptibility to amikacin, ceftazidime, ceftriaxone, meropenem, and ertapenem while demonstrating mixed susceptibility to co-trimoxazole, a trend that was also observed in our cohort [74]. Furthermore, beta-hemolytic *Streptococcus* isolates in our study showed 100% susceptibility to penicillin, erythromycin, co-trimoxazole, and rifampicin while demonstrating mixed resistance to tetracycline and clindamycin, a pattern similar to that reported by Torrens et al. [74]. Therefore, regional and global antimicrobial surveillance are of the utmost importance in order to better understand and combat the rise of multidrug-resistant pathogens.

In summary, our study showed *Staphylococcus aureus* and *Pseudomonas aeruginosa* as being major pathogens in chronic wounds, with distinct microbial compositions observed in HS compared to VU. We also found moderate antibiotic resistance, particularly among *S. aureus* and *P. mirabilis,* underscoring the need for personalized treatment strategies. The diversity of bacterial species suggests that wound microbiomes are highly patient-specific and also region-specific. These findings highlight the critical need for continuous surveillance of microbial populations and antibiotic resistance patterns in order to optimize the management of chronic wounds.

However, this study has several limitations that should be considered. First, the relatively small sample size and lack of diversity in the patient population may limit the generalizability of the findings to broader populations. Additionally, the cross-sectional design restricts the possibility to further observe changes in microbial populations over time. Using culture-based methods may have caused certain unculturable bacteria to be missed, and antibiotic resistance testing was limited to a standard panel, thus potentially underestimating the full spectrum of resistance profiles. Furthermore, the study did not include a control group of healthy individuals, which would have helped distinguish between pathogenic and non-pathogenic bacteria. Lastly, variations in antibiotic use and wound-care practices across patients could have influenced microbial diversity and resistance patterns.

## 4. Materials and Methods

We conducted a cross-sectional observational study examining patients diagnosed with hidradenitis suppurativa (HS) and venous ulcers (VU) who were admitted to a dermatology clinic in Bucharest, Romania, over nearly four years (January 2021–September 2024). The study cohort comprised 24 patients with HS and 88 with VU, all of whom presented with chronic wounds. The inclusion criteria required patients to have positive wound swab cultures, while the exclusion criteria eliminated cases with incomplete data or negative wound swab results. Wound colonization was confirmed through wound swabs and cultures conducted in a single laboratory. For each patient, we recorded demographic and clinical data, including age, gender, primary diagnosis, culture results, and antibiogram findings. Data were systematically collected using Microsoft Excel and subsequently analyzed using IBM SPSS Statistics version 23 for statistical evaluation. Also, the charts and tables represented in this paper were designed using Microsoft Excel. In addition, we used AI tools for text refining and styling and grammar improvements.

## 5. Conclusions

In conclusion, this study provides valuable insights into the microbial colonization patterns and antibiotic resistance in patients with hidradenitis suppurativa and venous ulcers. Our findings underscore the predominance of *Staphylococcus aureus* as the most common pathogen in both HS and VU, with distinct differences in bacterial species distribution between the two groups. Notably, *Pseudomonas aeruginosa* was identified exclusively in VU patients, while beta-hemolytic *Streptococcus* and *Corynebacterium amycolatum* were identified in HS cases only. Furthermore, the study highlights moderate antibiotic resistance among certain bacterial strains, including *S. aureus* and *Proteus mirabilis*, but no significant resistance patterns were detected across the entire cohort. Despite these findings, the study’s small sample size and the limited diversity of the patient population present constraints on the generalizability of the results. Further studies with larger and more heterogeneous groups are necessary to confirm these trends and improve the management of chronic wound infections, particularly in light of growing concerns over multidrug-resistant pathogens. Overall, continuous monitoring of microbial dynamics and resistance profiles in chronic wounds is crucial for optimizing treatment strategies and minimizing complications.

## Figures and Tables

**Table 1:** Summary Analysis of Antibiotic Resistance and Susceptibility Among Key Isolates.

Bacterial Species	N			Mean			Std. Deviation			Median			Percentile 25			Percentile 75		
	R	S	I	R	S	I	R	S	I	R	S	I	R	S	I	R	S	I
*Staphylococcus aureus*	45	45	45	1.64	6.11	0.04	1.708	2.014	0.208	2.00	7.00	0.00	0.00	5.00	0.00	3.00	7.00	0.00
*Proteus mirabilis*	7	7	7	2.29	7.14	0.29	1.380	1.952	0.756	2.00	7.00	0.00	1.00	6.00	0.00	4.00	9.00	0.00
*Pseudomonas aeruginosa*	24	24	24	0.88	4.50	2.67	2.232	1.216	1.465	0.00	5.00	3.00	0.00	3.25	3.00	1.00	5.00	4.00
*Enterococcus faecalis*	10	10	10	0.10	4.50	0.00	0.316	1.841	0.000	0.00	4.50	0.00	0.00	3.00	0.00	0.00	5.00	0.00
*Escherichia coli*	5	5	5	0.80	5.60	0.00	0.837	2.510	0.000	1.00	5.00	0.00	0.00	4.00	0.00	1.50	7.50	0.00
Beta-hemolytic *Streptococcus*	2	2	2	1.00	5.00	0.00	1.414	1.414	0.000	1.00	5.00	0.00	0.00	4.00	0.00	-	-	0.00

N = number of isolates; R = resistant; S = susceptible; I = intermediate.

**Table 2:** Antimicrobial resistance profile of *Staphylococcus aureus*.

Tested Antibiotic	R	S	I
Oxacillin	14/43 (32.6%)	29/43 (67.4%)	0/43 (0%)
Gentamicin	3/43 (7.0%)	40/43 (93.0%)	0/43 (0%)
Amoxicillin-Clavulanate	1/3 (33.3%)	2/3 (66.7%)	0/3 (0%)
Erythromycin	22/40 (55.0%)	18/40 (45.0%)	0/40 (0%)
Tetracycline	8/20 (40.0%)	12/20 (60.0%)	0/20 (0%)
Minocycline	1/22 (4.5%)	21/22 (95.5%)	0/22 (0%)
Clindamycin	23/43 (53.5%)	20/43 (46.5%)	0/43 (0%)
Levofloxacin	2/6 (33.3%)	2/6 (33.3%)	2/6 (33.3%)
Co-trimoxazole	0/43 (0%)	43/43 (100%)	0/43 (0%)
Linezolid	0/16 (0%)	16/16 (100%)	0/16 (0%)
Rifampicin	0/38 (0%)	38/38 (100%)	0/38 (0%)
Vancomycin	0/20 (0%)	20/20 (100%)	0/20 (0%)
Teicoplanin	0/12 (0%)	12/12 (100%)	0/12 (0%)
Tigecycline	0/2 (0%)	2/2 (100%)	0/2 (0%)

R = resistant; S = susceptible; I = intermediate. Results are shown as numerator/denominator, where the denominator represents the total isolates tested, excluding any missing data.

**Table 3:** Antimicrobial resistance profile of *Proteus mirabilis*.

Tested Antibiotic	R	S	I
Ampicillin	4/7 (57.1%)	3/7 (42.9%)	0/7 (0%)
Gentamicin	5/7 (71.4%)	2/7 (28.6%)	0/7 (0%)
Amikacin	0/6 (0%)	6/6 (100%)	0/6 (0%)
Amoxicillin-Clavulanate	0/7 (0%)	7/7 (100%)	0/7 (0%)
Tetracycline	1/1 (100%)	0/1 (0%)	0/1 (0%)
Cefuroxime	0/2 (0%)	2/2 (100%)	0/2 (0%)
Ceftazidime	0/6 (0%)	6/6 (100%)	0/6 (0%)
Ceftriaxone	0/6 (0%)	6/6 (100%)	0/6 (0%)
Ciprofloxacin	0/1 (0%)	0/1 (0%)	1/1 (100%)
Levofloxacin	1/5 (20.0%)	3/5 (60.0%)	1/5 (20.0%)
Co-trimoxazole	3/7 (42.9%)	4/7 (57.1%)	0/7 (0%)
Meropenem	0/5 (0%)	5/5 (100%)	0/5 (0%)
Ertapenem	0/5 (0%)	5/5 (100%)	0/5 (0%)
Colistin	1/1 (100%)	0/1 (0%)	0/1 (0%)
Tigecycline	1/1 (100%)	0/1 (0%)	0/1 (0%)
Piperacillin tazobactam	0/1 (0%)	1/1 (100%)	0/1 (0%)

R = resistant; S = susceptible; I = intermediate. Results are shown as numerator/denominator, where the denominator represents the total isolates tested, excluding any missing data.

**Table 4:** Antimicrobial resistance profile of *Pseudomonas aeruginosa*.

Tested Antibiotic	R	S	I
Oxacillin	1/24 (4.2%)	23/24 (95.8%)	0/24 (0%)
Gentamicin	0/24 (0%)	4/24 (16.7%)	0/24 (0%)
Amikacin	1/21 (4.8%)	20/21 (95.2%)	0/21 (0%)
Tobramycin	1/18 (5.6%)	17/18 (94.4%)	0/18 (0%)
Erythromycin	0/24 (0%)	1/24 (4.2%)	0/24 (0%)
Clindamycin	0/24 (0%)	1/24 (4.2%)	0/24 (0%)
Ceftazidime	1/21 (4.8%)	2/21 (9.5%)	18/21 (85.7%)
Ceftriaxone	0/24 (0%)	2/24 (8.3%)	0/24 (0%)
Levofloxacin	8/19 (42.1%)	2/19 (10.5%)	9/19 (47.4%)
Co-trimoxazole	0/24 (0%)	3/24 (12.5%)	0/24 (0%)
Linezolid	0/24 (0%)	1/24 (4.2%)	0/24 (0%)
Rifampicin	0/24 (0%)	1/24 (4.2%)	0/24 (0%)
Imipenem	1/20 (5.0%)	19/20 (95.0%)	0/20 (0%)
Meropenem	2/21 (9.5%)	18/21 (85.7%)	1/21 (4.8%)
Vancomycin	0/24 (0%)	1/24 (4.2%)	0/24 (0%)
Colistin	0/24 (0%)	18/24 (75.0%)	0/24 (0%)
Piperacillin Tazobactam	2/21 (9.5%)	2/21 (9.5%)	17/21 (81.0%)
Ceftazidime Avibactam	1/14 (7.1%)	13/14 (92.9%)	0/14 (0%)
Cefiderocol	0/24 (0%)	1/24 (4.2%)	0/24 (0%)
Ceftolozane Tazobactam	1/24 (4.2%)	0/24 (0%)	0/24 (0%)
Imipenem Relebactam	1/2 (50.0%)	1/2 (50.0%)	0/2 (0%)
Meropenem Vaborbactam	1/24 (4.2%)	0/24 (0%)	0/24 (0%)

R = resistant; S = susceptible; I = intermediate. Results are shown as numerator/denominator, where the denominator represents the total isolates tested, excluding any missing data.

**Table 5:** Antimicrobial resistance profile of *Enterococcus faecalis*.

Tested Antibiotic	R	S
Ampicillin	0/9 (0%)	9/9 (100%)
Gentamicin	1/10 (10%)	9/10 (90%)
Amikacin	0/1 (0%)	1/1 (100%)
Tobramycin	0/1 (0%)	1/1 (100%)
Ceftazidime	0/1 (0%)	1/1 (100%)
Ceftriaxone	0/1 (0%)	1/1 (100%)
Levofloxacin	0/1 (0%)	1/1 (100%)
Co-trimoxazole	0/1 (0%)	1/1 (100%)
Linezolid	0/2 (0%)	2/2 (100%)
Meropenem	0/1 (0%)	1/1 (100%)
Ertapenem	0/2 (0%)	2/2 (100%)
Vancomycin	0/8 (0%)	8/8 (100%)
Colistin	0/1 (0%)	1/1 (100%)
Teicoplanin	0/3 (0%)	3/3 (100%)
Tigecycline	0/3 (0%)	3/3 (100%)
Ceftazidime avibactam	0/1 (0%)	1/1 (100%)

R = resistant; S = susceptible. Results are shown as numerator/denominator, where the denominator represents the total isolates tested, excluding any missing data.

**Table 6:** Antimicrobial resistance profile of *Escherichia coli*.

Tested Antibiotic	R	S
Ampicillin	3/5 (60%)	2/5 (40%)
Gentamicin	0/5 (0%)	5/5 (100%)
Amikacin	0/1 (0%)	1/1 (100%)
Tobramycin	0/1 (0%)	1/1 (100%)
Amoxicillin-Clavulanate	1/5 (20%)	4/5 (80%)
Ceftazidime	0/2 (0%)	2/2 (100%)
Ceftriaxone	0/1 (0%)	1/1 (100%)
Levofloxacin	0/1 (0%)	1/1 (100%)
Co-trimoxazole	0/5 (0%)	5/5 (100%)
Meropenem	0/1 (0%)	1/1 (100%)
Ertapenem	0/1 (0%)	1/1 (100%)
Piperacillin tazobactam	0/4 (0%)	4/4 (100%)

R = resistant; S = susceptible. Results are shown as numerator/denominator, where the denominator represents the total isolates tested, excluding any missing data.

**Table 7:** Antimicrobial resistance profile of beta-hemolytic *Streptococcus*.

Tested Antibiotic	R	S
Penicillin	0/2 (0%)	2/2 (100%)
Erythromycin	0/2 (0%)	2/2 (100%)
Tetracycline	1/2 (50%)	1/2 (50%)
Clindamycin	1/2 (50%)	1/2 (50%)
Co-trimoxazole	0/2 (0%)	2/2 (100%)
Rifampicin	0/2 (0%)	2/2 (100%)

R = resistant; S = susceptible. Results are shown as numerator/denominator, where the denominator represents the total isolates tested, excluding any missing data.

## Data Availability

The data are available from the corresponding author upon reasonable request.
